# Risk factors for recurrent disease after resection of solitary fibrous tumor: a systematic review

**DOI:** 10.3389/fsurg.2024.1332421

**Published:** 2024-01-31

**Authors:** Johan Tolstrup, Anand Loya, Ninna Aggerholm-Pedersen, Louise Preisler, Luit Penninga

**Affiliations:** ^1^ Department of Surgery and Transplantation, Rigshospitalet, Copenhagen, Denmark; ^2^ Department of Pathology, Rigshospitalet, Copenhagen, Denmark; ^3^ Department of Oncology, Aarhus University Hospital, Aarhus, Denmark; ^4^ Department of Clinical Medicine, Copenhagen University, Copenhagen, Denmark

**Keywords:** solitary fibrous tumor, risk factor, prognosis, pathology, sarcoma

## Abstract

**Introduction:**

Solitary fibrous tumor (SFT) is a rare soft tissue tumor found at any site of the body. The treatment of choice is surgical resection, though 10%–30% of patients experience recurrent disease. Multiple risk factors and risk stratification systems have been investigated to predict which patients are at risk of recurrence. The main goal of this systematic review is to create an up-to-date systematic overview of risk factors and risk stratification systems predicting recurrence for patients with surgically resected SFT within torso and extremities.

**Method:**

We prepared the review following the updated Prisma guidelines for systematic reviews (PRISMA-P). Pubmed, Embase, Cochrane Library, WHO international trial registry platform and ClinicalTrials.gov were systematically searched up to December 2022. All English studies describing risk factors for recurrence after resected SFT were included. We excluded SFT in the central nervous system and the oto-rhino-laryngology region.

**Results:**

Eighty-one retrospective studies were identified. Different risk factors including age, symptoms, sex, resection margins, anatomic location, mitotic index, pleomorphism, hypercellularity, necrosis, size, dedifferentiation, CD-34 expression, Ki67 index and *TP53*-expression, APAF1-inactivation, TERT promoter mutation and *NAB2::STAT6* fusion variants were investigated in a narrative manner. We found that high mitotic index, Ki67 index and presence of necrosis increased the risk of recurrence after surgically resected SFT, whereas other factors had more varying prognostic value. We also summarized the currently available different risk stratification systems, and found eight different systems with a varying degree of ability to stratify patients into low, intermediate or high recurrence risk.

**Conclusion:**

Mitotic index, necrosis and Ki67 index are the most solid risk factors for recurrence. TERT promoter mutation seems a promising component in future risk stratification models. The Demicco risk stratification system is the most validated and widely used, however the G-score model may appear to be superior due to longer follow-up time.

**Systematic Review Registration:**

CRD42023421358.

## Introduction

**Figure 1:**
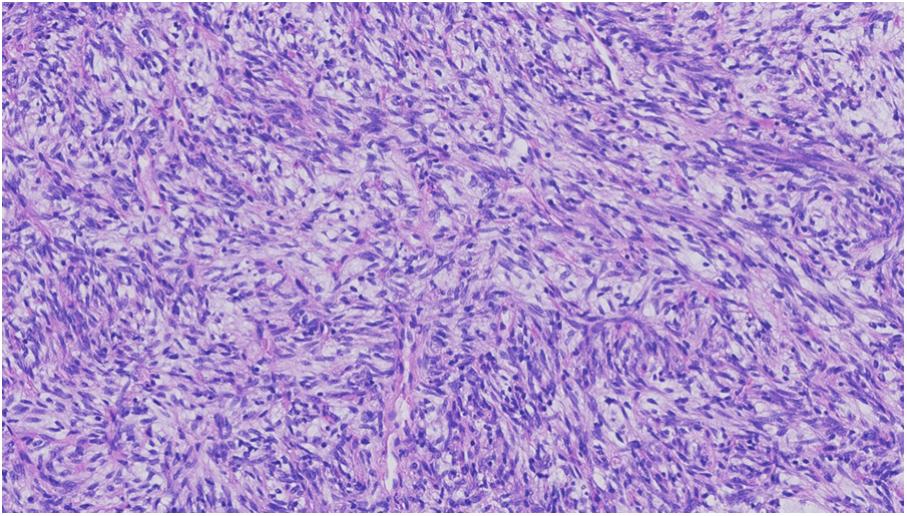
SFT with charcteristics “Patternless pattern” predominantly spindle cell morphology with cellular atypia (HE 22X).

**Figure 2:**
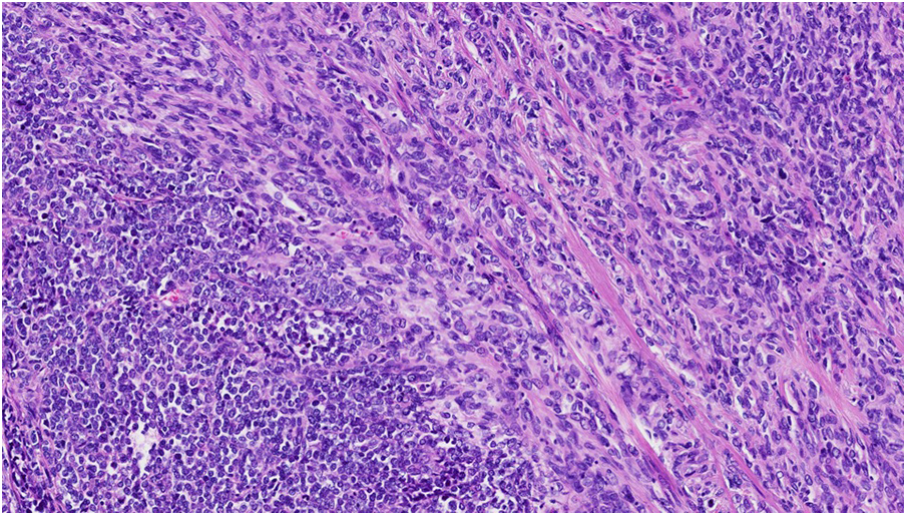
Malignant SFT with both spindle cell areas and round cell areas (HE 14X).

Solitary fibrous tumor (SFT) is a rare soft tissue tumor. Morphologically the cells typically appear with oval to spindle-shaped nuclei surrounded by scarce cytoplasm and intervening collagen fibres arranged in a “patternless” pattern (Figure 1). Different SFT variants such as giant-cell containing, dedifferentiated, myxoid, fat-forming and pleomorphic forms have been described. The final diagnosis of SFT is based on the immunohistochemical detection of a fusion between *NAB2::STAT6* genes, in practice by using STAT6 immunochemical stain (1, 2) (Figure 2).

The most common tumor location is within the thoracic cavity and abdomen, but SFT can be found throughout the whole body (3). Surgical resection with negative margins is the recommended treatment. SFTs can be benign or malignant, typically based on the criteria by England et al. (4), but even benign SFTs can metastasize, and this unpredictable nature poses a clinical challenge and questions the follow-up after treatment. Recurrence rates are varying and have been estimated to approximate 10%–20% (5, 6), but in studies with longer follow-up time recurrence rates of more than 30% have been reported (7).

Multiple risk factors have been proposed to predict which patients are at risk of recurrence (5, 8–11). In addition, numerous risk stratification systems (RSS) have been developed to predict recurrence risk. In an extra-meningeal cohort, Demicco et al. found age, size, necrosis and mitotic index to be predictive of recurrence (12), however Georgiesh et al. found, in their RSS, that mitotic index, necrosis and sex better identified the low-risk patients (11). Some models, like Diebold et al, developed a RSS specifically for pleural SFT and found mitotic index, size, Ki-67 index and necrosis to be the best predictive variables (10). Hence, there exist controversies regarding risk factors, and in addition, the development in molecular and genetic techniques has made it possible to investigate new potential risk factors for patients with SFT (13, 14). These factors create a need for an up-to-date systemic review of the current knowledge in this field.

## Methods

### Study design

This systematic review followed the PRISMA extension guidelines for systematic reviews (PRISMA-P). The protocol was registered in the Prospero Database with registration number: CRD42023421358.

### Participants

Inclusion criteria were: randomized controlled trials (RCTs), reviews, observational studies (*n*≥ 5) reporting on children or adults, who were treated for histologically confirmed SFT, and reported data on risk factors or potential risk factors for adverse outcome such as local recurrence, metastasis, reduced disease-free survival, disease-specific mortality, etc.

Also, we included studies assessing performance of risk-stratification models.

We excluded studies where patients were treated exclusively for SFT in CNS (and meninges) as well as in the oto-rhino-laryngology region, since these anatomic sites were out of scope for this systematic review. Studies where patients only received radio- or chemotherapy were also excluded.

### Search strategy

A systematic search was made in the following databases: PubMed, Embase, and the Cochrane Library. Furthermore, the WHO international trial registry platform and ClinicalTrials.gov were searched to identify ongoing studies. We restricted inclusion from the year 2000 until December 2022.

The search strategy was created with help from an information specialist. Search terms were: “Solitary fibrous tumor” and “hemangiopericytoma”. No efforts were made to find “grey” literature.

### Data extraction

References were screened by two researches (JT and LP), initially on title and abstract level, to exclude studies clearly out of scope. Disagreements were solved by discussion. A second screening process was carried out, and the full-text articles were read in order to make a final inclusion of studies. Again, consensus was obtained after discussion. Data was extracted by predefined data-charts: title, author, year of publication, demographic data, setting, follow-up, results regarding risk-factors or risk stratification models.

### Risk of bias

Due to the fact that included studies only comprised retrospective cohorts and case-series, the “JBI Critical Appraisal Tool” was found appropriate to assess risk of bias. It contains 10 questions and assesses internal validity, risk of selection and information bias as well as the quality of reporting of results. This tool has been used in various studies (15).

Briefly, question 1, 2 and 3 address the inclusion of patients, and if the condition is measured in a standardized and valid way. Question 4 and 5 address whether or not the inclusion was consecutive and complete. Question 6, 7 and 8 address reporting of demographics, clinical information and follow-up. Question 9 addresses the geographic location of the clinic in which the study is carried out. Question 10 addresses the statistical methods used.

## Results

A total of 3,289 studies were initially identified, 829 duplicates were removed, and 2,460 studies were eligible for title and abstract screening. A total of 2,323 studies were excluded leaving 137 studies for full text assessment. Due to inappropriate study design (reviews, conference abstracts, editorial comments, etc.), or studies which did not full-fill the inclusion criteria (no prognostic data or risk factors included) another 63 studies were excluded. Finally, we identified 7 relevant references from other reference lists, and included these in the total number of 81 included studies. Inclusion is summarized in Table 1.

**Table 1:** Inclusion process.

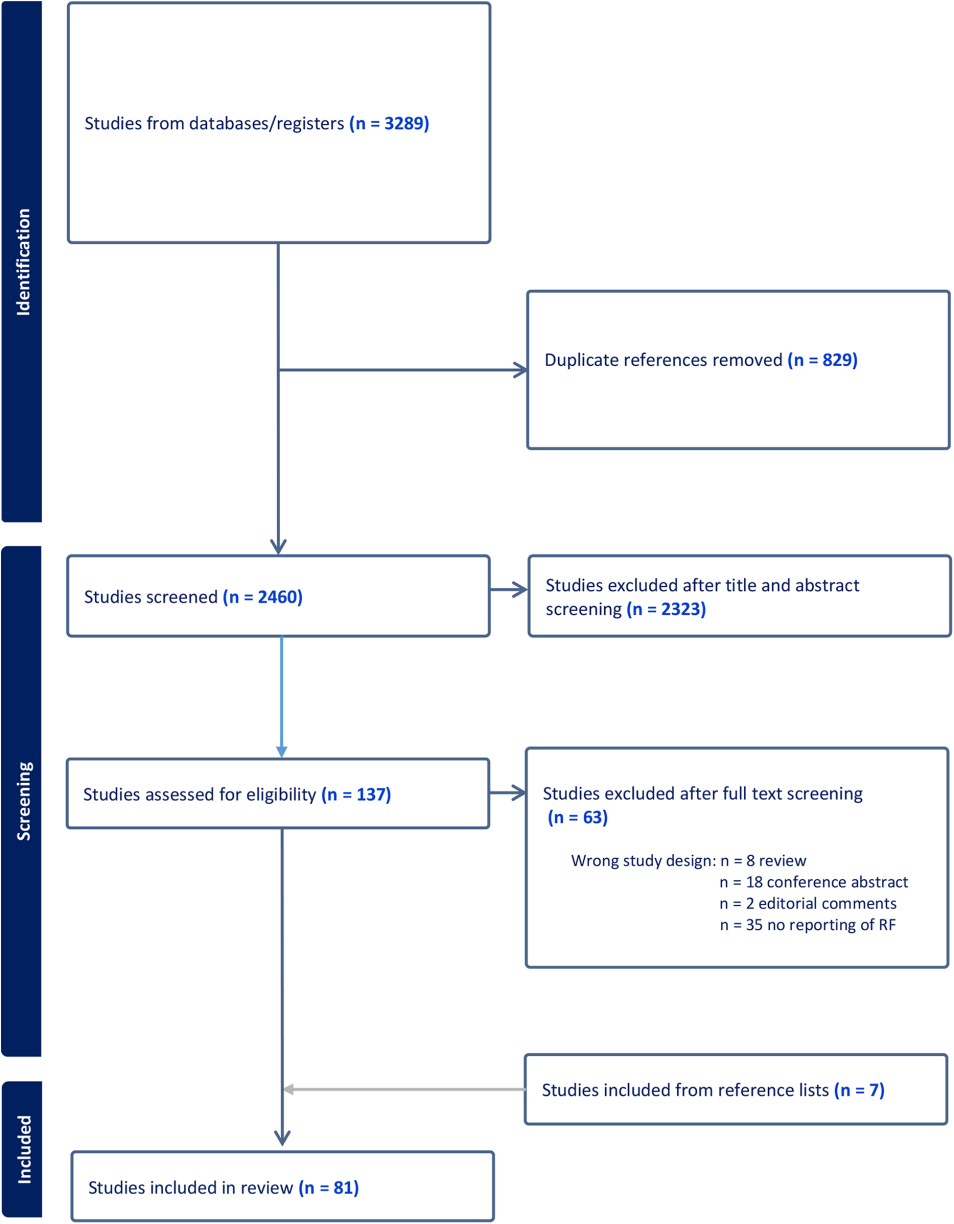

### Study characteristics

We did not find any randomized controlled trials, nor did we find prospective cohort studies, thus all included studies were retrospective cohort studies. The numbers of cases in the included studies ranged between 11 and 549 (16, 17). Median and mean patient age ranged from 50 to 67 years (18, 19) and 57% of studies had a slight predominance of female patients. Follow-up time was not clearly reported for 14 studies, the remaining 67 studies reported mean or median follow-up time between 12 months to 168 months (20, 21).

In all 81 studies, patients were diagnosed with SFT either by biopsy or based on resection specimens, and almost all patients were treated with surgical resection. The vast majority of studies included patients with primary, localized SFT, however a minority of case-series included locally advanced or metastatic SFT. Twenty-nine studies reported SFT at any anatomic site of the body, twenty-five pleuro-pulmonary or in the chest/thorax (mediastinum, lung and pleura), eleven extra meningeal, three extra-thoracic and extra-meningeal, two in the urogenital tract, one in bones, one in extremities, one in retroperitoneum, one in the mesentery and liver, one in the retroperitoneum and pelvis, and one in pelvis.

Relapse from SFT was typically measured as either time to local recurrence or metastasis [disease-free survival (DFS), recurrence-free survival (RFI) event-free survival (EFS)]. Overall survival (OS) and disease-specific death (DSD) were also calculated for some studies.

### Clinical and demographic risk factors

#### Age

Many studies have investigated age as an independent risk factor for adverse outcome after resected SFT. As expected, age is often correlated to inferior OS (8, 12), however, Demicco et al. found a significant correlation between higher age and metastasis in two large cohorts of patients with extra-meningeal SFT (5, 12), and this is why age was included in their risk stratification model. The largest cohort to date, with 613 SFT cases, also found reduced disease-free survival (DFS) for patients above 51 years, however this study was characterized by missing data, i.e., 70% of the SFT patients lacked proper staging (3). Furthermore, Ghanim found positive associations between age ≥59 and reduced event free survival (EFS) in a cohort of intrathoracic SFT (22). Opposite to this, numerous studies did not find such correlations (23–34), and recently a Norwegian group with a long follow-up time (median of 84 months), did not find association between age and recurrence free interval (RFI) (11).

#### Symptoms

Only a minor fraction of studies has investigated the prognostic role of symptoms vs. no symptoms. We only found studies without association between symptoms and adverse outcome (25, 33, 35).

#### Sex

Most studies find no relation between sex and risk of recurrence or reduced OS (3, 22, 28, 30, 31, 33, 36–38), however, one study by Reisenauer et al. found worse OS for male patients in a univariate analysis (39). Interestingly, Georgiesh et al. found that male gender was associated with increased risk of late recurrence (11), and thus added male gender to their risk score (G-score).

#### Resection margins

Surgical removal of the SFT is the cornerstone in the treatment, but the significance of radical resection is still not clear. Most series, however demonstrate adverse outcome (LR, metastasis, shorter event-free survival, etc.) after positive resection margins (6, 12, 19, 22, 40, 41). One of the largest cohorts with 303 SFT patients found a marked increased risk of local recurrence (HR = 10.0) in the cohort with positive margins (12). Surprisingly, a large study with 162 patients with extra meningeal SFT, did not find positive resection margins (R0 vs. R1) as risk factor of neither OS, local recurrence or metastatic recurrence in univariate or multivariate analysis (8), and neither did Deanna Wand et al. find any significant association between R0 vs. R1 resection and local recurrence, metastasis or OS in their cohort of 59 SFT patients (36).

#### Anatomic location

The most frequent location for SFT is believed to in thorax followed by the abdomen/retroperitoneum (3). Numerous studies have found anatomic location to be a prognosticator for recurrence, however results are conflicting. Gholami et al. found location to be an independent predictor for recurrence and disease-specific death, and in their cohort of 219 patients, thoracic SFTs had the highest risk of local recurrence (5- and 10-year cumulative risk of 10% and 18%, respectively, compared to 4% and 7% for the total population consisting of SFT throughout the body). Regarding metastasis, SFTs in the abdomen/retroperitoneum had the highest risk with 10-year cumulative risk of 27% compared to SFT in thorax and in the head-neck region where 10-year cumulative risk was 16% and 15%, respectively (38). Also, Cranshaw et al. found intraabdominal, retroperitoneal and pelvic SFTs to have the highest risk of local recurrence (42). Wilky et al. found extra thoracic SFTs to be independently associated with recurrence (26), and O'Neil also found higher rate of malignancy in extra-thoracic SFTs (43). Luo et al. also found extra thoracic SFTs to be more aggressive (28), and in accordance with these results Akaike et al. found the extra thoracic location to be associated with lower disease-free survival rate (44). The largest cohort to date found SFT in thorax/abdomen/pelvis to be favorably associated with DFS compared to SFT in CNS or head-neck region (3). Salas et al. found SFT in the limbs to be associated to increased risk of metastasis in both uni- and multivariate analysis (8). Finally, 4 studies did not find any correlation between anatomic location and risk recurrence (7, 31, 36, 41).

### Pathological risk factors

#### Mitotic index

Number of mitosis [≥4 mitosis/high-power fields (HPFs)] has traditionally been a central criteria in the distinction between malignant and benign SFT (4). Indeed, mitotic index seems to be higher in malignant SFT, and it is found to be prognostic for recurrence or metastasis regardless the anatomic location (3, 5–8, 10–12, 14, 17, 22, 23, 25, 26, 29, 32, 36, 37, 39–42, 44–52).

Three studies did not find mitotic index to be a significant prognostic risk factor (31, 38, 53).

#### Pleomorphism

Pleomorphism is often referred to as variation in shape and form of the nuclei in the tumor. We found 8 studies where pleomorphism was found to be a risk factor of adverse outcome (6, 10, 11, 37, 40, 44, 54, 55). Four studies did not find any significant prognostic value of pleomorphism (5, 25, 49, 56).

#### Hypercellularity

Hypercellularity can be seen as excessive amount of crowded cells and overlapping nuclei with minimally intervening collagen (39), and this feature has been investigated for its prognostic value. We found 7 studies which proved hypercellularity to be significantly associated with recurrence or other measures of adverse outcome (6, 37, 39, 40, 49, 52, 56), however, 5 studies could not find similar results (5, 11, 27, 30, 32).

#### Necrosis

We found 15 studies which found a significant higher risk of recurrence, metastasis or reduced OS when necrosis was present in the tumor (5, 6, 10, 11, 19, 32, 36, 37, 39, 40, 48, 49, 54, 55, 57). Demicco et al. added necrosis to their original 3-item score, thus making it better to identify low risk patients (32). However, 7 studies did not find necrosis to be prognostic of adverse outcome (25, 27, 30, 31, 41, 45, 57).

#### Size

Whether tumor size is a risk factor for tumor recurrence is a subject of debate, and results are very conflicting. Demicco and Gholami both found that tumor size was an independent risk factor for disease-specific death and risk of metastasis, respectively (5, 38), which explains the inclusion of tumor size in the original 3-tiered risk assessment model by Demicco et al. Also, a number of other studies found similar correlations (10, 12, 17, 25, 28, 32, 37, 39, 41, 49, 55, 56). Opposite to the above mentioned studies, we found 16 studies which could not find any significant correlation between size and recurrence (7, 8, 21, 26, 27, 29–31, 33, 34, 36, 46, 48, 57, 58). Surprisingly, we even found an inverse correlation between tumor size and DFS and OS in a study based on 243 patients with resectable extra-meningeal, extra-pleural SFT (6). Of note, a series with pleural SFTs by Woodard et al. included nine giant SFTs with a mean diameter of more than 20 cm, and none of these experienced recurrence (48).

#### Dedifferentiation

Morphologically, dedifferentiation in SFT is described as an abrupt transition from areas with conventional SFT to areas resembling a high-grade sarcoma (59). Dedifferentiation is very rare and the available evidence is scarce, however some studies indicate a worse prognosis for patients with dedifferentiated SFT. In a case-series from 2009, three out of eight patients with dedifferentiated SFT died from their disease (60), and in a case-series of 10 dedifferentiated SFT, seven of ten patients died because of their disease within a median of 73 months from diagnosis. Also, Yamada et al. found dedifferentiation to be an independent risk factor of recurrence (61). Finally, Sugita et al. found dedifferentiation to be significantly associated with worse 5 year metastasis-free survival, however only 2 of 43 patients had dedifferentiated SFT in their study (31).

### Immunohistochemical risk factors

#### CD34

The expression of CD34 glycoprotein on the cell membrane is common in SFT, yet not specific, when diagnosing SFT (62), and some studies have investigated its prognostic potential. Franzen et al. found no difference in CD34 expression between malignant and benign SFT, and no prognostic value of this marker (25). In accordance with these results, DeVito et al. did not find CD34 status to predict OS in a cohort of 82 patients (46). Diebold et al. graded CD34 staining from weak to strong (4 categories), but found no correlation to adverse outcome (10). Interestingly, a minor fraction of SFTs are CD34 negative, and in a study by Lahon et al. CD34 negativity was significantly associated with recurrence of malignant SFT (21). Similarly, Dermawan et al. also found that CD34 negative SFTs were more likely to metastasize than CD34 positive tumors (20).

#### Ki67-index

The Ki67 protein is present on the cell nucleus, and it reflects the proliferative potential of the tumor cells, thus high percentage of Ki67 is known to be a prognosticator in many malignant conditions. Sugita et al. found that the Ki67 LI (labeling index) ranged from <1% to 72%, and they divided their samples in low (Ki67 < 1%) with 35% of the patients, intermediate (Ki67 1%–10%) with 56% of the patients and high (Ki67 ≥ 10%) with 9% of the patients. Patients with high Ki67 had a significantly higher risk of metastasis within 5 years of surgery and furthermore, the authors substituted mitotic index with Ki67-index in Demiccos RSS, and found it to be potentially superior (31). We found more studies in which high Ki67 was associated with adverse outcome (63, 64), however Ki67 cut-off values differed from ≥2% (39), ≥5% (30), ≥10% (10, 19, 65) and ≥12% (25).

#### TP53 expression

Mutations in *TP53* may lead to dysfunction of the tumor suppressor gene P53. Traditionally, *TP53* status is measured by immunohistochemistry (IHC), but DNA-sequencing, PCR and other techniques are also available. Machado et al. found a low prevalence of *TP53* mutations (15 out of 97 samples), and no clear correlation to adverse outcome was found, but *TP53* was more common in high risk SFT (14). Park et al. found *TP53* immuno-positivity to be significantly associated with local recurrence and metastasis (13), which is in accordance with findings from Schirosi, Akaike and Rodriguez-Gonzalez (37, 44, 63), however these results were disputed by others (10, 57, 66).

#### APAF1

APAF1 (apoptotic protease-activating-factor1) is involved in the process of apoptosis, and some researchers have proposed, that inactivation of APAF1 could be involved in malignant transformation of SFT. Park et al. found a correlation between APAF1 inactivation and malignancy, but not with local recurrence or metastasis (13). Machado et al. found no correction between APAF1 status (positive or negative) and clinical outcome (14).

### Molecular risk factors

#### TERT promoter mutation

Mutations in the TERT promoter region may promote aggressive behavior in SFT, and it is present in about 20%–40% of SFTs (14, 67). In a large series with 172 patients Demicco demonstrated an increased risk of metastasis when TERT promoter mutation was present (HR = 2.9), however no correlation to OS or disease-specific death was found (67). Bahrami and Akaike found likewise TERT promoter mutation to be associated with lower event-free survival (44, 68). Park and Lin however, only found TERT promoter mutation to be associated with malignancy, but not with local recurrence or metastasis (13, 69). Bianchi studied 41 patients with SFT in the extremities and found TERT promoter mutation to be associated with risk of metastasis (57). Salguero-Aranda found that TERT promoter mutation was associated with reduced progression-free survival and OS (66). Machado et al. found TERT promoter mutation was more frequent in patients with high and intermediate risk stratification, thus speculating that this feature could be particularly useful in risk stratification of the “intermediate” group of SFT patients (14). Finally, a recent study by Krsková et al. fount TERT promoter mutation to be associated with malignant behavior, but not strictly with risk of recurrence (64).

#### NAB2::STAT6 fusion variants

In 2013 two research groups discovered the *NAB2::STAT6* gene-fusion to be diagnostic for SFT (65, 70), and now more than 40 different fusion variants have been discovered. Many studies have investigated whether these different fusion variants have different malignant potential.

We found two studies which proved *NAB2::STAT6* fusion variants to have a clear prognostic significance. Barthelmess discovered 12 different fusion variants in 52 patients. *NAB2ex4::STAT6ex2* (*n* = 25), *NAB2ex6::STAT6ex16* (*n* = 7), and *NAB2ex6::STAT6ex17* (*n* = 4), were the most frequent events. They found significantly higher risk of recurrence in the *NAB2ex6::STAT6ex16/17* group. Georgiesh studied 39 patients and found 12 different fusion variants. They divided the fusion variants into two groups based on the length of the STAT6 gene, the so-called *STAT6-TAD* and *STAT6-full*. Patients with *STAT6-TAD* had an increased risk of local recurrence, distant recurrence and OS in the univariate analysis (71).

Park et al. discovered 3 different fusion variants in 68 cases: 1b (*NAB2*ex4::*STAT6*ex2) in 56%, 2a (*NAB2*ex6::*STAT6*ex16) in 13%, 2b (*NAB2*ex6::*STAT6*ex17) in 6%, but found no association to malignant potential (13). Machado found the most common fusion variants to be *NAB2-exon4::STAT6-exon2* followed by *NAB2-exon6::STAT6-exon16/17*, but failed to find them to be predictive of aggressive behavior (14). Akaike found 7 types *of NAB2::STAT6* fusion variants in 40 cases, the most frequent being *NAB2exon4::STAT6exon2*. They found *NAB2exon4::STAT6exon2-3* to be associated with less aggressive phenotype, but correlation with lower DFSR was not present (44). Likewise, seven other studies with SFT from various anatomic sites, did not find significant correlation between fusion variants and adverse outcome (57, 61, 64, 72–76).

### Risk stratification models

SFT is an unpredictable tumor, making it notoriously difficult to estimate recurrence risk and plan surveillance. Therefore, many different research groups have made great efforts to develop risk stratification systems (RSS), which have clearly improved prognostication for patients with primary SFT (Table 2). As seen from the examples below, RSS are typically based on various combinations of clinical and histomorphological variables which have been identified as independent risk factors in multivariate analyses.

**Table 2:** Risk stratification systems.

Risk stratification score	Anatomic site	Prognostic factors
Georgiesh et al. (11)	Extra-meningeal	- Gender - Mitotic index - Necrosis
Demicco et al. (12)	Extra-meningeal	- Mitotic index - Size - Age - Necrosis
Demicco et al. (5)	Extra-meningeal	- Mitotic index - Size - Age
Salas et al. (8)	Extra-meningeal	- Mitotic index - Age - Anatomic site
Tapias (2012)	Pleural	- Pleural origin (parietal or visceral) - Morphology (pedunculated or sessile) - Size - Hypercellularity - Necrosis/hemorrhage - Mitotic index
Diebold et al. (10)	Pleural	- Mitotic index - Size - Ki67 index (MIB-1) - Necrosis
De Perrot et al. (9)	Pleural	- Hypercellularity - Mitotic index - Pleomorphism - Hemorrhage - Necrosis - Invasion - Morphology (pedunculated or sessile)
Pasquali et al. (6)	Extra-thoracic	- Mitotic index - Cellularity - Pleomorphism
	Extra-meningeal	

RSS can be separated into three different groups, according to the anatomic location of the SFT from which they are developed:

We identified four RSS developed and validated in extra-meningeal SFT:

Three-variable risk score from Demicco (original D-score) including age, size and mitotic rate (5). Four-variable risk score from Demicco including age, size, mitotic rate, necrosis (modified D-score) (32). Three-variable risk score from Salas 2017 (separated in Salas overall survival (Salas^OS^), Salas metastasis (Salas^MET^), Salas local recurrence (Salas^LR^)) including mitotic rate, age and anatomic site (8). Three-variable G-score by Georgiesh based on male sex, necrosis and mitotic count (11).

We found three RSS developed and validated in pleura-pulmonary SFT:

The six-variable risk score by Tapias based on pleural origin, morphology, size, hypercellularity, necrosis/hemorrhage, mitotic rate (49). The four-variable risk score by Diebold based on mitotic rate, size, Ki67 index (MIB-1) and necrosis (10). Finally, de-Perrot who staged from 1 to 4 based on 6 different histological malignancy signs (hypercellularity, mitotic rate, pleomorphism, hemorrhage, necrosis, invasion) and morphology (pedunculated or sessile) (9).

We found one RSS based on extra meningeal and extra pleural SFT, namely a study by Pasquali, they made a scoring system based on: mitotic rate, cellularity and pleomorphism (6).

#### Comparison of RSS

Georgiesh collected data from 318 patients with primary, extra meningeal SFT. G-score could be calculated for 211 patients, 23% low risk, 43% intermediate risk and 34% high risk. The modified D-score was used to calculate risk for 224 patients, 56% low risk, 26% intermediate risk and 18% high risk. Salas^OS^ were calculated for 248 patients, 36% low risk, 44% intermediate risk and 19% high risk. There was a surprisingly poor correlation between the three models. The modified D-score performed best to identify high-risk patients, however the G-score was best to identify low-risk patients (7). These results were in accordance with previous work from Georgiesh et al, where 6 and 7 patients from the low-risk groups in the revised D-score and Salas^OS^ score developed recurrence of disease, respectively. Only one patient from the G-score low-risk group developed recurrence. Of interest, many of the recurrences occurred several years after treatment, in fact median time to recurrence was >5 years (11).

Demicco performed a comparison between their own modified D-score, Salas^OS^, Salas^MET^, Salas^LR^ and Pasquali on a cohort of 303 SFT patients. Modified D-score, Salas^MET^ and Salas^OS^ were better than Pasquali to predict the risk of metastasis and RFS, however none of the RSS were able to significantly predict local recurrence. The modified D-score was best to identify the patients at lowest- and highest risk (12).

Ricciardi tested the Tapias-score, the modified D-score and de Perrot RSS and found that Tapias better predicted OS and DFS compared to the others in a cohort of 34 SFT patients with metastatic, pleuro-pulmonary SFT (19).

Reisenauer found that both the original and modified D-score, Tapias and de Perrot predicted progression-free survival, but only the D-scores and Tapias predicted OS, with a slightly better discrimination in the modified D-score (39).

A recent study of patients with intraabdominal SFT compared the modified D-score, Salas and Pasquali. None of the RSS were able to predict LR, however, the modified D-score and Salas^OS^ had the best performance (54).

Silverwood tested the revised D-score and Pasquali-score on a small cohort of 12 patients with extra-thoracic and extra-meningeal SFT, and found the Pasquali model to perform better than the D-score (77).

Bellini collected a patient cohort with 107 pleural SFT. They found Tapias and Diebold to be independently associated with tumor recurrence, however, de Perrot was not. Tapias had the highest reliability with a highly significant *p*-value (*p* < 0.0001) (29).

Diebold et al. developed their own scoring system for SFT, and found it superior to the Tapias score. As much as 44% of the patients in their cohort could not be scored according to de Perrot due to missing data (10).

Finally, Tapias validated their own score on a population of 113 pleural SFTs. They found a score sensitivity of 78% and specificity of 74% compared to 100% and 92% in the development cohort. However, they outperformed both the scoring system by de Perrot, and the classic malignancy criteria by England (52).

### Risk of bias

Overall, only retrospective studies were identified, and no prospective studies have been performed, which increases the risk of bias. We found that nearly all studies reported well-established inclusion criteria (histological diagnosis of SFT), however many studies did not perform an extra (central) pathological confirmation of the samples.

The vast majority of studies did not report whether the inclusion was consecutive or complete, usually the authors denounced that a number of SFT-cases were identified, typically from a pathological database with no further details.

In general, the studies thoroughly reported demographics, clinical information and follow-up, and most studies also provided estimates of “missing data”.

We only found scarce information on geographic characteristics on the clinic or clinics responsible for the treatment. Often, it was stated, that it was a tertiary centre.

All studies had a proper description about the applied statistical methods, however, with varying level of detail.

## Discussion

We have provided a systematic, up-to-date review regarding risk factors and risk stratification systems after treatment of SFT. We found 81 retrospective studies investigating both clinical, demographic, histological, immunohistochemical and molecular risk factors. The most reliable prognostic marker was the mitotic index, typically measured as ≥4 mitosis/high-power fields. Furthermore, the presence of necrosis appeared to be a solid risk factor. Other histological markers, such as pleomorphism and hypercellularity were generally regarded as signs of malignancy (4), but results were not clear in this review. Possibly, this might be due to low numbers of included patients in the cohorts and failure to reach statistical significance. Another weakness in the histological assessment of tumor tissue, is the risk of inter-observer differences. This is why some authors have explored the possibility to replace mitotic index with Ki67 LI in Demiccos' RSS, thus making measures of proliferative potential more objective (31). In this review we found elevated K-67 LI to be a clear risk factor for recurrence.

Surgical resection of SFT is the best treatment option, and many studies find, that a radical resection (R0) was associated with a better prognosis. Surprisingly, some studies did not find such associations, which may reflect the more aggressive nature of the tumor, or simply, that the cohorts lacked statistic power.

New molecular techniques have been applied in investigations of SFT, and in our review the most promising item was the TERT promoter mutation. Several studies found an association with either risk of recurrence or other malignant characteristics, and of particular interest, was the finding that TERT promoter mutation might ease risk stratification of patients who have intermediate risk of recurrence (14). In 2013 it was discovered, that *NAB2::STAT6* mutation was diagnostic for SFT, yet often the STAT6 staining was used as a surrogate marker (78). This invention is obviously extremely useful in the diagnostics of this rare and complex tumor, but there is no consensus regarding its prognostic value. More research is needed to elucidate this question.

Risk stratification of patients with SFT is also debated, and we found eight different RSS. The most validated RSS's are the models by Demicco (32, 34, 36), and they are the most widely used (12, 24, 39, 54). The revised D-score has more advantages. It is based on age, mitotic rate, size and necrosis, variables that are typically part of a histological report, thus making it easy to use. Furthermore, it can be used for SFT in all extra-meningeal sites, making it more universally applicable than for instance the model by Tapias (pleural) or Pasquali (extra-meningeal and extra-pleural). Nevertheless, the G-score seems to be a very promising tool as well, including male sex, necrosis and mitotic count, making it likewise easily calculated. It was published in 2020 (11), and validated in 2022 in a very large multinational cohort with promising results (7). The indisputable strength in the G-score is the long follow-up time (median 84 months) which is important, since SFT is able to relapse after several years, even after 15–16 years from initial treatment (38). More studies are needed clarify which RSSs are superior.

We did not find any RSS incorporating molecular findings, a possible future approach could be integration of TERT promoter mutation. It might be interesting to see if proteomics can be of any help in triaging SFT's into different categories. But so far, there haven't been any study utilizing proteomics.

### Limitations

This review has some weaknesses. The included studies are all retrospective cohorts with great heterogeneity and an inherent risk of selection-bias. Also, SFTs are treated at tertiary centers from which these publications proceed, and this may cause a selection bias towards more advanced and potentially aggressive SFTs. Furthermore, some studies include SFTs removed 30–40 years ago enhancing the risk of a wrong diagnosis, especially since the majority of patients in these studies were included before the discovery of *NAB2::STAT6* gene-fusion in 2013. These reservations make it difficult to draw firm conclusions and recommendations. Publication-bias may also influence the results of this review, favoring publication of significant associations.

Initially, our ambition was to describe all risk factors or potential risk factors, however we had to omit a few. For instance, we encountered a study investigating fibrinogen (22), microRNA (79) or hemorrhage (30), and due to very scarce data, we chose not to describe these in detail.

Finally, there is a risk that all relevant studies may not be identified and included in this review. Even though we developed a thorough search strategy, strictly followed the PRISMA guidelines, and two authors selected studies, both the search strategy and screening process may lead to inappropriate exclusions.

## Conclusion

Several risk factors are known to predict recurrence after surgical resection of SFT. In this systematic review based on 81 retrospective studies, we found mitotic index, necrosis, KI67 index and possibly TERT promoter mutations to be the most valid risk factors. Of the numerous published risk stratification systems, the modified Demicco score is the most validated and widely used, however the G-score seems promising too. Even though, some studies did not find radical resection (R0) to be important for the prognosis, the corner-stone in treatment of SFT remains radical surgical resection.

## Data Availability

The original contributions presented in the study are included in the article/Supplementary Material, further inquiries can be directed to the corresponding author.
